# An Evidence-Based Guide for Medical Students: How to Optimize the Use of Expanded-Retrieval Platforms

**DOI:** 10.7759/cureus.10372

**Published:** 2020-09-11

**Authors:** Cyrus A Pumilia, Spencer Lessans, David Harris

**Affiliations:** 1 Medicine, University of Central Florida College of Medicine, Orlando, USA; 2 Medical Education, University of Central Florida College of Medicine, Orlando, USA

**Keywords:** spaced repetition, retrieval, optimization, anki, medical education, retention, knowledge acquisition, evidence-based learning, expanded-retrieval, active learning

## Abstract

Recommendations have been made for improving medical education based on the available evidence regarding learning. Traditional learning methods in medical education (e.g. reading from textbooks) do not ensure long-term retention. However, expanded-retrieval studying methods have been shown to improve studying efficiency. Using evidence-based practices to optimize an expanded-retrieval platform has the potential to greatly benefit knowledge acquisition and retention for medical students. This literature review was conducted to identify the best practices of expanded-retrieval platforms.

Themes within learning that promote knowledge gain and retention include presentation of related categorical information, schema formation, dual-coding, concrete examples, elaboration, changes in text appearance, and interleaving. Presentation of related categorical material together may mitigate retrieval-induced forgetting (RIF). Spaced retrieval helps to reinforce schema formation by solidifying the framework the individual students form when learning the material. Dual-coding improves learning by creating more neural pathways. Multiple concrete examples can be compared by students to see their respective differences, highlighting the true underlying principle. Variation in text appearance is most useful during the initial, short-term inter-study intervals. Interleaving is a theme where different topics are combined in the same study session and is unpopular with students but shown to be successful. Students’ subjective competency ratings of new material are largely inaccurate. More in-depth processing and learning methods that give off a sense of lower competency are actually associated with improved long-term retention.

Expanded-retrieval platforms should utilize these evidence-based components of learning to increase knowledge gain and retention within all fields of medical education.

## Introduction and background

Memory can be trained to seemingly impossible levels [1], indicating that there may be potential for improvement of how knowledge is acquired in medical school. Accordingly, there have been recommendations for improving medical education based on the available evidence in cognitive science regarding learning [2, 3]. As technology continues to expand, more web-based materials are being used by medical students, many of which being expanded-retrieval platforms. Expanded-retrieval platforms are platforms commonly used for content review where the interval between testing of subject matter is gradually increased. These platforms allow students to study information in increasing inter-study intervals (increasing time between the testing or studying of subject matter) with retrieval-based practice. Expanded-retrieval may also be called “spaced repetition,” “expanding rehearsal,” “graduated intervals,” “repetition spacing,” and “spaced retrieval.”

Previous studies have demonstrated that learning in medical education does not often ensure long-term retention [4-6]. An experiment performed in residents showed that implementation of a tutorial on clinical guidelines improved mean knowledge scores from 50% pre-test to immediate post-test scores of 76%. However, scores then dropped to approximately half of what was gained between three and eight days and to an unmeasurable gain in retention at fifty-five days [4]. Even students that have been shown to benefit from this testing effect fall back to normal forgetting characteristics if not re-exposed to the material [7]. In the study conducted by Kerfoot in 2010, retention at one week was improved, but at six months, there was no increased retention [8].

Expanding retrieval has been shown to improve studying efficiency, netting similar test scores at the end of a study period as equal-spacing but with lower amounts of total repetitions [9]. Students given immunology and physiology material showed more improvements in testing after trials of expanding intervals rather than with equal intervals when the total number of repetitions are held constant [10]. It seems that, when the number of repetitions are held constant, spacing of repetitions is more effective than cramming them or spreading them equally. This has also been shown in urology residents using a web-based course [7].

Active retrieval is an effective learning tool in and of itself [8], typically producing greater returns than re-studying [11]. Subjects learned paragraph readings better when some words were slightly blocked out, forcing subjects to continue reading via a process called “generative retrieval” [12]. Many expanded-retrieval platforms use a feature called “cloze deletions” based on the process of generative retrieval where users can create flashcards that hide words of a sentence. Generative retrieval was used for learning of cardiac anatomy in medical students and residents and was shown to be more effective over standard studying methods [13].

Multiple studies have recommended the use of expanded-retrieval platforms for medical students [14, 15]. The recommendations are made in accordance with how these platforms are used, but much of the current research points to future technology. The question remains as to how expanded-retrieval platforms can currently be implemented in medical education.

## Review

Methods

A PubMed search was conducted of appropriate terms, including all relevant iterations of expanded-retrieval: “Spaced AND repetition”, “Expanding AND rehearsal”, “Graduated AND intervals”, “Repetition AND spacing”, “Repetition AND scheduling”, “Spaced AND retrieval”, “Expanded AND retrieval”, “retrieval AND practice AND learn”, “retrieval AND practice”, “medical AND student AND recall”, “interleaving AND learning”, “flashcard AND learn”, “dual AND coding”, “dual AND coding AND learn”, and “Retrieval AND induced AND forgetting AND education.” The literature search was conducted in June 2018. 

Articles were included based on relevancy to the population in question and the application in question. For articles to be included, they had to meet the following criteria: they must have been published in English and they had to study or analyze spaced repetition methods in the process of learning for the purposes of education. Studies that looked at spaced repetition methods outside the field of education were not included. For example, studies concerning the use of expanded-retrieval for the purposes of brain injury rehabilitation were excluded, and studies concerning the use of interleaving for the purposes of motor skill acquisition were similarly excluded. 

**Figure 1:**
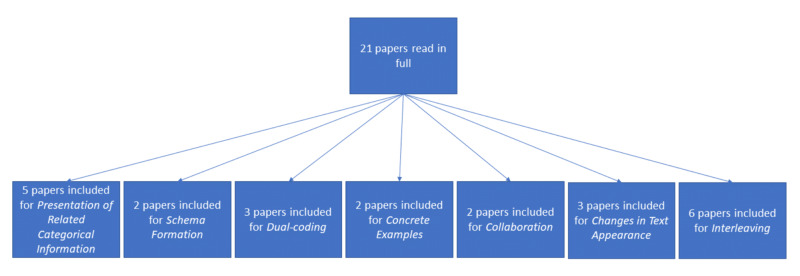
Article Inclusion Process

In total, 21 papers were found to be relevant and read in full by the authors. There was no blinding done when analyzing whether the papers were to be included or not. The papers were included if they met the inclusion criteria above and fell under one of the themes of expanded-retrieval platforms (Figure 1). This narrative review was not designed to be a systematic analysis of the use of expanded-retrieval platforms within medical education. Rather, it analyzes and summarizes the current literature to determine what themes of expanded-retrieval platforms are evidence-based. 

Results and analysis

Themes that can be implemented within expanded-retrieval platforms include presentation of related categorical information, schema formation, dual-coding, concrete examples, elaboration, changes in text appearance, and interleaving. The role of each of these themes in learning and expanded-retrieval programs will be discussed below.

Presentation of Related Categorical Information

**Figure 2:**
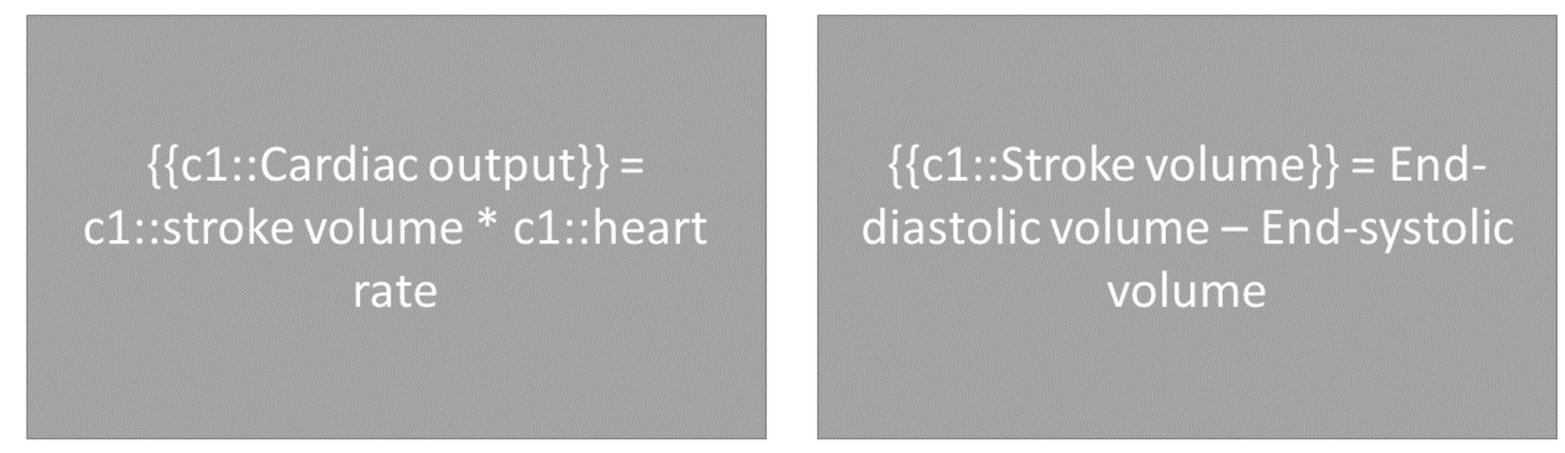
Presentation of related information: testing recognition of different concepts within the same field {{c1::}} is used in spaced repetition programs to add 'Cloze deletions' where the phrase contained in the brackets is hidden for the user to recall.

Presentation of related categorical material together may mitigate retrieval-induced forgetting (RIF) [16, 17]. When a category of information is learned, and only a portion of said category is presented for retrieval at a later date, the memory of the presented information is strengthened while the memory of unpresented information is weakened, even if retrieval is unsuccessful [18]. The most significant effects of RIF are seen when closely related facts are tested (Figure 2) [17]. However, information forgotten through RIF was learned more quickly than other not-yet-seen information that was presented, thereby reversing the effects of RIF [19]. Subjects with more anxiety have been shown to have some protection against RIF [20], which may have implications for RIF in proximity to important examinations that increase anxiety. Expanded-retrieval platforms can minimize RIF by allowing for viewing of material that is not explicitly tested but is present upon presentation of related material, such as supporting information on flashcards. In doing so, medical students will be presented with a greater amount of information than through conventional studying methods.

Schema Formation

**Figure 3:**
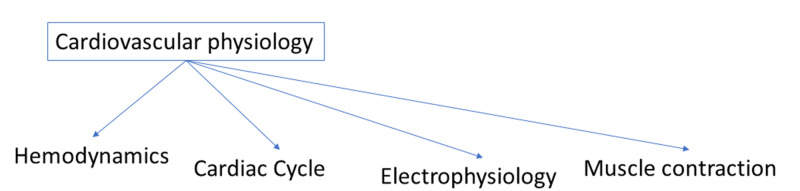
Schema formation: flashcards can be organized by concepts grouped inside a larger field to allow for the formation of schemas

Schema formation, a student's "framework" in which they fill knowledge, has been shown to be important for long-term retention. Schema reinforcement seems to be important for reinforcement in learning [21]. Spaced retrieval helps to reinforce schema formation by solidifying the framework the individual students form when learning the material (Figure 3) [22]. As a result, it may be important to implement the different framework types that work best for the different individual learners into the expanded-retrieval program.

*Dual-Coding* 

**Figure 4:**
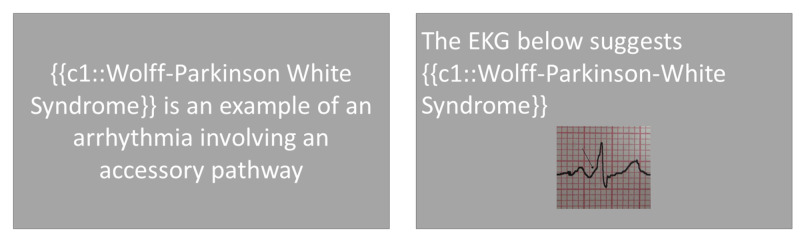
Dual-coding: both text and images can be used to enhance learning of a topic {{c1::}} is used in spaced repetition programs to add 'Cloze deletions' where the phrase contained in the brackets is hidden for the user to recall.

Dual-coding, the use of multiple sensory inputs to learn a specific set of information, also can improve retention. It has been shown to be effective in healthcare fields [23] though its main effects have been shown for vocabulary and foreign language learning [24]. It is thought to improve learning by creating more neural pathways, thereby increasing the chance of pathway utilization during an attempted recall. Multisensory learning seems to facilitate learning via one sensory modality as well and may be used as a gauge of proper schema formation [21]. Expanded-retrieval programs can make use of this by combining both verbal and visual information electronically when presenting material (Figure 4).

Concrete Examples

**Figure 5:**
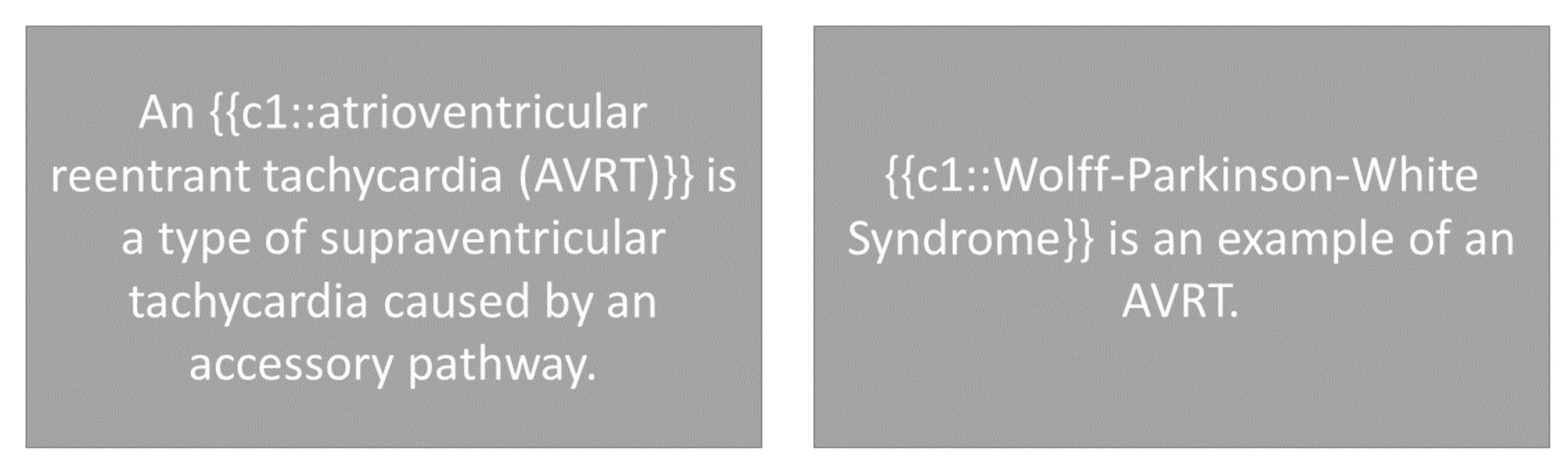
Concrete examples: specific examples of concepts elaborated in the previous flashcards can be given {{c1::}} is used in spaced repetition programs to add 'Cloze deletions' where the phrase contained in the brackets is hidden for the user to recall.

Concrete examples also help students understand complex concepts and recognize patterns. Giving concrete examples for concepts can help students connect concepts (Figure 5) [25], but may pose a risk for the disregarding of concepts in exchange for simply following the specific example [26]. Accordingly, students should utilize multiple examples that go along with their expanded-retrieval platform’s concept-based presentations. Multiple examples can be compared to see their respective differences, highlighting the true underlying principle. More examples may help to ensure that an accurate pattern is seen and the simple memorization of one example does not occur.

Elaboration

**Figure 6:**
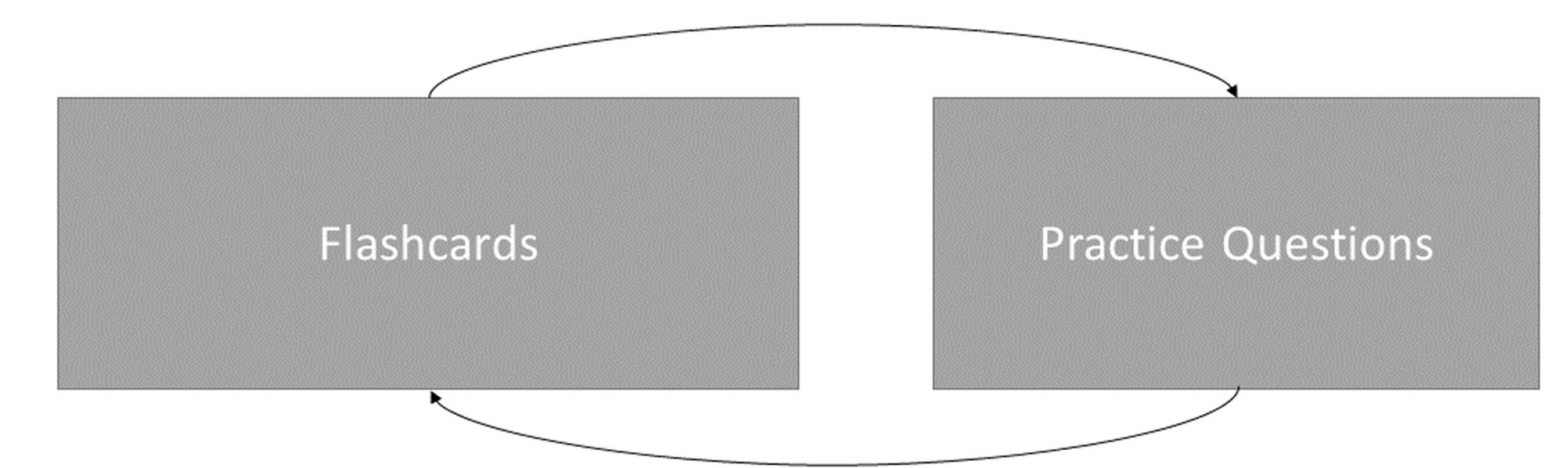
Elaboration: flashcards can be utilized with other resources to consolidate learning

Elaboration, an act of allowing the mind to freely question a topic, has been shown to be effective for learning. Central to elaboration is the process of overcoming uncertainty, a process that is crucial for learning [27]. Accordingly, students may utilize elaboration when expanded-retrieval fails, and they are forced to re-study a topic. Elaboration simply consists of a student letting the mind wander about the topic, self-testing, and finding answers to the questions that come to mind (Figure 6). This may require temporary exit of the expanded-retrieval platform, a potential drawback to utilizing these platforms. An important note to make is that elaboration must be made with review of information known to be accurate, as elaboration techniques may lead students astray to false understandings of material [28].

*Changes in Text Appearance* 

**Figure 7:**
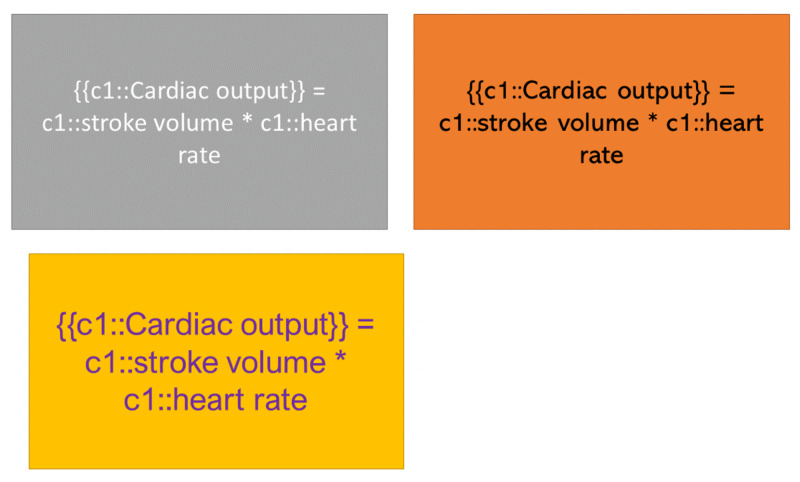
Changes in text appearance: changing the appearance will prevent rote memorization {{c1::}} is used in spaced repetition programs to add 'Cloze deletions' where the phrase contained in the brackets is hidden for the user to recall.

Changes in text appearance, including inversion of words [29] and hard to read fonts [30], result in improved retention (Figure 7). The effects are thought to be due to activation of deeper processing of the material. While this effect is attainable outside an expanded-retrieval platform, this variation impact on learning can certainly translate to within these platforms. For example, a student can change the size of the computer window used, altering the shape of the text appearance. A student may also change the colors of the program if that platform allows so.

Variation seems to be most useful during the initial, short-term inter-study intervals, whereas at longer inter-study intervals, it may actually inhibit further retention [31], thought to be due to the interference with the initial framework, or schema, that the memory was stored in. This inhibitory effect was greatest for recall testing rather than for recognition testing. Accordingly, while variation in appearance may initially help in the learning process, students should be weary of too much variation at long inter-study intervals.

Interleaving

**Figure 8:**
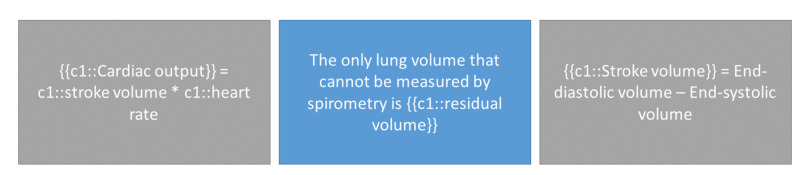
Interleaving: mixing in unrelated topics can help aid comprehension {{c1::}} is used in spaced repetition programs to add 'Cloze deletions' where the phrase contained in the brackets is hidden for the user to recall.

Interleaving is the act of mixing topics during a study session (Figure 8). Though students report beliefs that interleaving would be counterproductive [32], a large body of research supports its use. Interleaving has been shown to be effective for both visual stimuli [33] and for textual learning [34]. It must be noted that interleaving has been shown to be ineffective for studying subjects that are too far apart, such as anatomy and Indonesian language [35]. Interleaving improves learning by allowing discriminative-contrast, as learning of different concepts in the same study session allows for the direct comparison between the two, which can improve future performance [36]. In interleaving, difficulty seems higher. It appears that the more difficulty the learner perceives, the stronger the long-term retention effects [37]. In support of this contrast, subjects rated massing (combining similar topics when studying) as more productive than interleaving, though interleaving was objectively more productive [33-34].

It is also important to reduce reliance on students’ subjective competency ratings as short-term perceptions have been shown to be largely inaccurate [38]. More in-depth processing and learning methods that give off a sense of lower competency are associated with improved long-term retention [38]. Retrieval is a way to alleviate the false sense of knowledge gained while studying that has been shown to be inaccurate [39]. Effective interventions for improving subjective measures can be done by spaced retrieval platforms by consistently presenting the material again and again, the interval being modified with the personal knowledge of failed retrieval or for desired increased frequency at times where greater repetition is desired (i.e. if an examination is soon).

There is data that suggests that expanding-retrieval, recall, and/or spacing is not the best choice for certain purposes. For example, the positive effects of expanding-retrieval are likely not seen from a single study session [40]. Also, a study in residents with a web-based, equally-spaced interval program showed no improvement in subsequent test performance unless the subjects were first-year interns [41], which shows that expanding-retrieval platforms may not be relevant to all medical education populations. Furthermore, testing with feedback was shown to be superior to simple, spaced study in residents on medical knowledge [42].

We also know that testing enhances learning [43] and improves access to marginal knowledge even without feedback [40, 44]. Testing, given that the answer choices are close and plausible (i.e. “competitive”), but clearly have a correct or incorrect component (so that the incorrect answers can be seen and identified), can foster recall/further learning of both correct and incorrect answer choices [45]. Some studies have shown lack of benefit of multiple choice over retrieval [44], which seems to be due to the answer choices not facilitating proper comparison of information. Testing may even have a generalizable effect to material not tested upon. A generalized testing effect was seen in language learning where testing improves retention of other, recently-studied language terms that were not tested [46]. Unfortunately, testing takes more time than expanded-retrieval and integrating testing with spacing is more difficult than simple expanding-retrieval platforms. Nonetheless, this sheds light on the scope of efficacy and the need for other learning tools to be integrated into a student’s learning appropriately.

## Conclusions

The current evidence provides guidance for students to optimize their use of expanded-retrieval platforms. These platforms can utilize evidence-based components of learning such as presentation of related categorical material together, schema formation, and interleaving, among the others mentioned to increase knowledge gain and retention. All of these themes have been shown to improve knowledge acquisition. Expanded-retrieval platforms, such as Anki, are already commonly used by medical students. The integration of peer-reviewed research into expanded-retrieval platform algorithm optimization may be a direction for future research.
